# Flotillin2 Expression Correlates with HER2 Levels and Poor Prognosis in Gastric Cancer

**DOI:** 10.1371/journal.pone.0062365

**Published:** 2013-05-02

**Authors:** Zhi Zhu, Jinou Wang, Zhe Sun, Xuren Sun, Zhenning Wang, Huimian Xu

**Affiliations:** 1 Department of Surgical Oncology, Department of General Surgery, The First Hospital of China Medical University, Shenyang, China; 2 Department of Pathology, Shengjing Hospital of China Medical University, Shenyang, China; 3 Department of Digestion, The First Hospital of China Medical University, Shenyang, China; University of Pécs Medical School, Hungary

## Abstract

**Objective:**

Flotillin gene is known as a tumor promoter or suppressor, depending on the tumor type or tumor stage. We aimed to investigate the clinical significance of flotillin2 protein expression in gastric cancer.

**Methods:**

We examined flotillin2 and erbB2 levels in tissue microarray of 282 gastric cancer samples and analyzed the association between flotillin2 levels, clinicopathologic factors and prognosis. The regulation of erbB2 by flotillin2 was examined with flotillin2 siRNA–transfected gastric cancer cells.

**Results:**

Flotillin2 partially co-localized with erbB2 at the plasma membrane as detected by confocal microscopy, levels of erbB2 were reduced after flotillin knockdown in SGC-7901 cancer cells, and the expression of flotillin2 was positively correlated with that of erbB2. In non-neoplastic gastric mucosa, flotillin2 was not expressed in the epithelial compartment. In gastric cancer, positive staining of flotillin2 was shown in 129 (45.7%) of 282 cases, also, it was significantly associated with a Lauren grade, histologic type, lymphovascular invasion and tumor location. Moreover, survival analysis showed that flotillin2 expression was an independent prognostic factor of poor survival (p<0.001).

**Conclusions:**

These results indicate that a positive correlation exists between flotillin2 and erbB2 expression levels, flotillin2 maybe involved in the stabilization of erbB2 at the plasma membrane, flotillin2 is significantly correlated with cancer progression and poor prognosis in gastric cancer.

## Introduction

Many plasma membrane proteins are known to be involved in cell proliferation, cell adhesion, cell motility and tumor cell invasion, accounting for more than two thirds of currently known drug targets. The cell membrane and associated proteins are of substantial interest with regard to various aspects of tumor, from carcinogenic and metastatic mechanisms to molecular diagnosis and therapeutics [1]. Flotillins are associated with vesicular invaginations of the plasma membrane, and act as regulators of signal transduction [2]. Among flotillin family members, there are two different flotillin genes (flotillin1 and 2), flotillin2 protein expression has been reported in some human cancer cell lines and tumor samples [3], [4], [5], [6]. Since flotillin2 directly interacts with signaling molecules such as receptors, kinases, adhesion molecules and G proteins, it acts as a tumor modulator through the regulation of cell proliferation, differentiation, apoptosis, adhesion and invasion [4].

Overexpression of erbB2 (human epidermal growth factor receptor 2), also known as HER2, is membrane-associated tyrosine kinase that can lead to the activation of cellular signal transduction systems, resulting in the cellular transformation and cell proliferation events associated with cancer, such as breast cancer, ovarian cancer and gastric cancer [7], [8], [9]. High expression level of erbB2 has been significantly correlated with increased tumor invasion, metastasis, resistance to chemotherapy, and poor prognosis of patients [10]. Recent studies demonstrated that flotillin2 protein, among other functions, was involved in endocytic mechanisms and cellular trafficking processes, and was found to be in a molecular complex with erbB2 [11]. To understand whether stabilization of erbB2 at the plasma membrane is mediated by flotillin2 and their clinical significance, we carried out comparative membrane proteomic analysis of human gastric cancer.

In this study, we describe flotillin2 and erbB2 expression levels are positively correlated on a cellular level as well as in gastric cancer tissue and we show that erbB2 is internalized and degraded by a mechanism upon flotillin2 depletion. Furthermore, the clinicopathologic significance of flotillin2 was further evaluated using archival tissue specimens and statistical analysis. We found that flotillin2 is an independently prognostic factor, also a potential novel biomarker for lymph node metastasis. Our data will facilitate an understanding of gastric cancer carcinogenesis and mining biomarkers for the diagnosis and treatment of this disease.

## Materials and Methods

### Tissue Samples and Follow-Up

A total of 282 patients who had surgery for gastric cancer between January 2006 and December 2009 at the First affiliated hospital of China Medical University was selected for this study. All patients-derived specimens were collected and archived under protocols approved by the Institutional Review Boards of the First affiliated Hospital China Medical University. The diagnosis was confirmed by at least two pathologists and staging was based on pathological findings according to the 7th American Joint Committee on Cancer guidelines. The median duration of follow-up was 51 (range, 5–78) months.

The 282 patients who underwent gastroctomy were subjected to close clinical observation, including chest and abdominal CT imaging, CEA level, and blood testing at 2- to 3-month intervals and a yearly gastroscopy. Overall survival (OS) rates were defined as the interval from the initial surgery to clinically or radiologically proven recurrence or metastasis and death, respectively. The end date of the follow-up study for conducting the analysis was June 29, 2012.

### Ethics statement

Ethical approval for this research was obtained from the Research Ethics Committee of China Medical University, China. All patients providing tumor tissue as well as normal gastric tissue samples signed a consent form prior to surgical removal of the gastric carcinoma to allow for this research to be undertaken.

### TMA construction and immunohistochemistry

Hematoxylin and eosin (H&E)-stained slides were screened for optimal tumor tissue and noncancerous tissue adjacent to tumor (at least 2 cm from the tumor) and TMA slides were constructed with a tissue manual arraying instrument. Two cores were collected from each formalin-fixed, paraffin-embedded (FFPE) gastric cancer tissue sample and from each normal gastric mucosa sample using a 1.0-mm diameter punch instrument. Samples from the same patient were spotted next to each other to ensure similar reaction conditions for the normal and tumor tissue of that patient. Immunohistochemical Analysis was performed on FFPE samples as described previously using an Envision kit (Dako Cytomation, Glostrup, Denmark) [12]. Pressure cooker mediated antigen retrieval was performed in citrate buffer (pH 6.0) for 7 min. Sections were incubated with 1 200 dilution of anti-flotillin2 (Santa Cruz, sc-25507) and erbB2 (sc-33684) overnight at 4°C, and then incubated with goat anti-mouse or anti-rabbit Envision System Plus-HRP (Dako Cytomation) for 30 min at room temperature. After rinsing three times in PBS for 10 min each, the sections were incubated with DAB for 1 min, counterstained with Mayer hematoxylin, dehydrated, and mounted.

### Evaluation of Immunohistochemical Staining

Immunoreactivity was evaluated independently by two researchers who were blinded to patient outcome. The evaluation was based on the staining intensity and extent of staining. Staining intensity for flotillin2 and erbB2 was scored as 0 (negative), 1 (weak), 2 (moderate), and 3 (strong). Staining extent was scored as 0 (0%), 1 (1–25%), 2 (26–50%), 3 (51–75%), and 4 (76–100%), depending on the percentage of positive-stained cells. The sum of the staining intensity and the staining extent scores was used as the final staining score. The specimens were divided into three groups according to their overall scores: 0–1, negative, 2–4, weak positive, and 5–7, strong positive.

### FISH analysis for gastric cancer cells

Amplification of the erbB2 gene was determined by dual-colour FISH method using the Passvision HER2 DNA probe Kit (Vysis Inc. Downers Grove, IL, USA) according to the manufacturer's protocol. The HER2-Spectrum Orange probe contains a DNA sequence specific for the erbB2 human gene locus and hybridizes to region 17q11.2–q12 of human chromosome. The CEP 17 (chromosome enumeration probe 17) used as a control contains alpha-satellite DNA. The nucleus was counterstained with 40, 6-diamidino-2-phenylindole (DAPI). The nude mice of gastric cancer model (SGC-7901, MGC-803 and BGC-823) were made as described before [13], [14]. The tumor slides were observed under BX60 fluorescence microscope equipped with digital camera (DP50) (Olympus, Tokyo, Japan), and the images were captured with Viewfinder software. A cell was considered to show amplification when a definite cluster or more than 10 signals for erbB2 was found.

### Cell culture and transfections

Gastric cancer cell lines SGC-7901 (moderately differentiated), MGC-803 and BGC-823 (poorly differentiated) were all purchased from the cell bank of Chinese Academy of Sciences. Cancer cell lines were maintained in RPMI 1640 (Hyclone, Logan city, USA) supplemented with 10% FBS. All the cell lines were in a 5% CO2 humidified atmosphere at 37°C. For siRNA transfection, cells were seeded out in six-well plates at a density of 2×10^5^ cells/well, 24 h before transfection, siRNAs targeting non-overlapping parts of the mRNA sequence were used for flotillin2. For the depletion of flotillin2, a pool of two different siRNA with the sequences targeting construct GAGGUUGUGCAGCGCAAUU and GGAUGAAGCUCAAGGCAGA [15], the cells were seeded out without antibiotics, grown for 24 h and transfected by using Lipofectamine transfection reagent (Invitrogen) according to the manufacturer's procedure. After 4 h of transfection, the medium was changed to complete growth medium containing serum and antibiotics, and the cells were grown for 3 days before experiments were started.

### Immunofluorescence, Western blot and qRT-PCR

Cells were washed twice with cold PBS and lysed on ice in RIPA buffer with protease inhibitors and quantified by BCA method. 50 µg Protein lysates were resolved on 6% SDS polyacrylamide gel, electrotransferred to polyvinylidene fluoride membranes (Millipore, Bedford, MA) and blocked in 5% nonfat dry milk in Tris-buffered saline (pH = 7.5). Membranes were immunoblotted overnight at 4°C with anti- flotillin2/erbB2 polyclonal antibodies as IHC described above, respectively, then followed by their respective secondary antibodies. Signals were detected by enhanced chemiluminescence (Pierce, Rockford, IL). For Immunofluorescence, the binding of primary antibody was visualized by TRITC/FITC-conjugated goat anti-mouse IgG antibody, and the slides were then examined by a confocal laser scanning microscope.

PCR amplifications for quantification of flotillin2, erbB2 and GAPDH mRNA in cells were done in a LightCycler system (Roche Applied Science) using the LightCycler FastStart DNA Master SYBR Green I kit (Roche Diagnostics). In brief, a master mixture was prepared on ice, containing 1 µl of complementary DNA, 2 µl of LC DNA Master SYBR Green I mix, 50 ng of primers, and 4 mM MgCl2. The amplification conditions for 40 cycles consisted of denaturation at 95°C for 10 s, annealing at 65°C for 10 s, and extension at 72°C for 10 s. The products were then subjected to a temperature gradient from 68 to 95°C at 0.1°C/s, with continuous fluorescence monitoring to produce melting curves of the products. The expression levels were normalized to erbB2 mRNA expression.

### Statistical analysis

The χ^2^ test or Fisher's exact test for proportion was used, as appropriate, to analyze the relationship between flotillin2 and erbB2 expression and clinicopathological variables. The survival rates were calculated by the Kaplan–Meier method and the differences between the survival curves were examined by the log-rank test. Univariate Cox proportional hazards regressions were applied to estimate the individual hazard ratio (HR). The significant variables in the univariate analyses (P<0.05) were then put into the multivariate analysis. The HR with 95% confidence interval (CI) was measured to estimate the hazard risk of individual factors. P<0.05 was considered to be statistically significant. Analyses were performed using the SPSS statistical software program version 19.0 (SPSS Inc., Chicago, IL).

## Results

### Expressions of flotillin2 and erbB2 in gastric cancer

**Figure 1:**
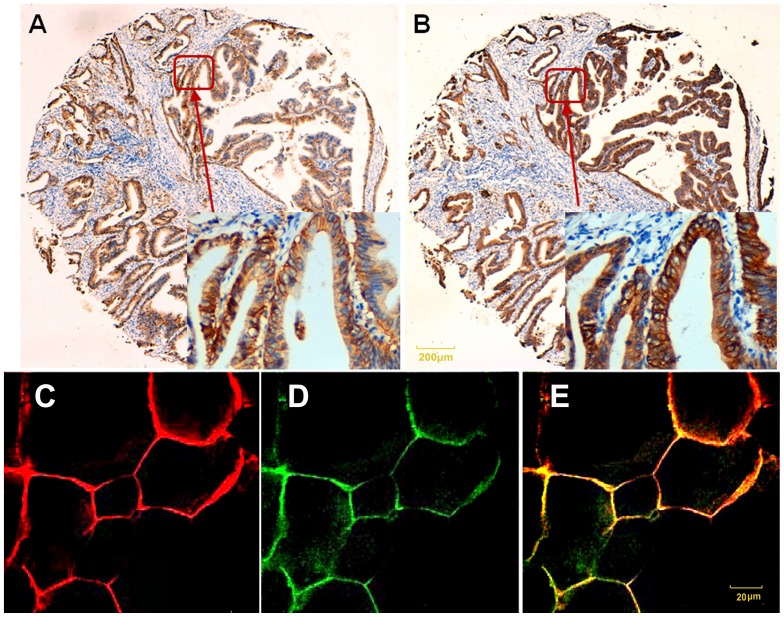
Correlation between flotillin2 and erbB2 expression, flotillin2 co-localize with erbB2 at the plasma membrane. Representative protein expression of (A) flotillin2 and (B) erbB2 in a tissue microarray core was taken from a gastric carcinoma. SGC-7901 gastric cancer cells were stained with anti-erbB2, anti-flotillin2 antibody respectively, the stained antibody was visualized by TRITC/FITC-conjugated goat anti-mouse IgG antibody, and the slides were then examined by a confocal laser scanning microscope. TRITC (erbB2) is shown as red (C), FITC (flotillin2) as green (D), and the merge of green and red becomes yellow (E).

**Figure 2:**
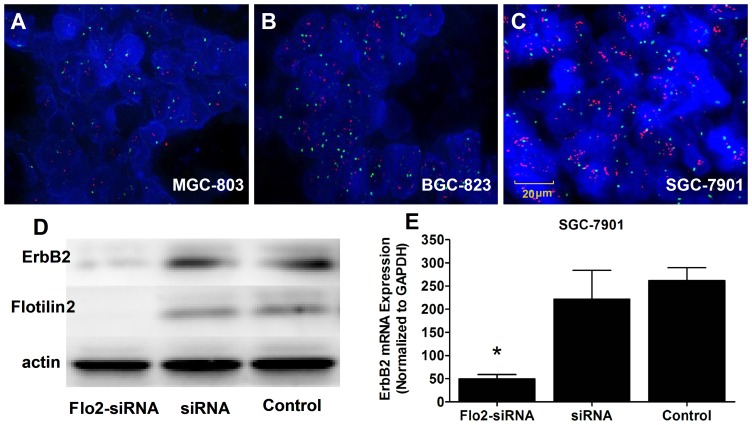
Knockdown of flotillin-2 resulted in a stronger reduction of erbB2. Amplification of HER2 gene was determined by FISH. HER2 gene amplification in a cluster pattern (red) was observed in (A) MGC-803, (B) BGC-823 and (C) SGC-7901 gastric cancer cells, HER2 over expression in human SGC-7901 cell. (D) Western blot analysis and (E) Quantitative real-time PCR were accessed for erbB2 and flotillin2 in SGC-7901 cell line after knockdown of flotillin-2. The expression status of flotillin2 is highly concordant with that of erbB2, siRNA was blank vector, β-Actin was used as internal control.

Stained sections of tissue microarrays (TMA) of 282 tissue cores were graded for their cytoplasmatic immunohistochemical staining intensity against flotillin2 and erbB2 protein. The 282 readable samples included 282 gastric carcinoma, 181 peri-carcinoma and 101 samples of normal gastric epithelium tissue. Tumor cells showed typical diffuse membrane staining of flotillin2 can be found in gastric carcinoma (Figure 1a) with the similar location of erbB2 (Figure 1b), in non-neoplastic gastric mucosa, flotillin2 and erbB2 were both not expressed. Positive staining of flotillin2 was shown in 129 (45.7%) of 282 specimens, erbB2 was 59 (20.9%). Amplification of erbB2 gene was determined by dual-colour FISH of xenografts in nude mice. ErbB2 gene amplification in a cluster pattern (red) was observed in MGC-803, BGC-823 and SGC-7901 cells, erbB2 overexpression only in human SGC-7901 gastric cancer cell (Figure 2a-c).

### Association between the expressions of flotillin2 with erbB2

We analyzed the cellular localization of erbB2 and flotillins in SGC-7901 gastric cancer cell. We could show by confocal microscopy that flotillin2 partially co-localize with erbB2 at the plasma membrane (Figure 1a-e). Our data shows that the expression of flotillin2 do has a significant correlation with that of erbB2 (p<0.001) in the immunohistochemical tissue array analysis (Table 1). In addition, interaction between flotillins and erbB2 could be demonstrated by immunoblot (Figure 2d) and quality RT-PCR experiments (Figure 2e), knocking down of endogenous flotillin2 resulted in pulling down of erbB2. We next analysed the impact of flotillins on the internalization and subsequent degradation of erbB2. As described below, erbB2 and her2 also have many relationships of clinicopathological factors in common.

**Table 1:** Flotillin2 expression in relation to the status of erbB2 expression.

	Flotillin2 positive	Flotillin2 negative	p value
erbB2			
Positive	43(38.6%)	16(61.4%)	<0.001*
Negative	86(72.9%)	137(27.1%)	

Figures in parentheses are percentages, % is within ErbB2

* p<0.05 considered to be statistically significant.

### Association between flotillin2 and clinicopathological factors

In this study, 282 gastric cancer patients with sufficient primary tumors materials and follow-up time were available, whose tissues were collected over the last 6 years. Table 2 gave the descriptive statistics for parameters measured for these patients. The median duration of the follow-up was 54 months (range 9–78 months). The 5-year overall survival (OS) rate was 48.2%.

**Table 2:** Correlation of Flotillin2 and erbB2 expression with clinicopathologic parameters in gastric cancer.

Variables	Cases	Flotillin2		P value	erbB2		P value
		−	+		−	+	
Gender		153	129	0.688	223	59	0.019
male	205	113	92		68	9	
Female	77	40	37		155	50	
Age(years)				0.901			0.556
≤65	182	98	84		142	40	
>65	100	55	45		81	19	
Size				0.006*			0.079
≤4 cm	135	85	50		113	22	
>4 cm	147	68	79		110	37	
Location				0.474			0.275
Lower	217	118	99		29	10	
Middle	21	12	9		15	6	
Upper	39	22	17		179	38	
Entire	5	1	4		0	5	
Macroscopic Type				0.231			0.202
Early stage	31	24	7		29	2	
Borrmann I	4	3	1		3	1	
Borrmann II	20	12	8		19	1	
Borrmann III	199	104	95		149	50	
Borrmann IV	28	10	18		23	5	
Histologic type				<0.001*			<0.001*
Differentiated	124	86	38		111	13	
Undifferentiated	158	67	91		112	46	
Lauren grade				0.001*			<0.001*
Intestinal	137	91	46		119	18	
Diffuse	145	62	83		104	41	
T Stage				0.001*			0.544
T1	31	18	13		26	5	
T2	50	29	21		29	6	
T3	181	99	82		137	44	
T4	20	7	13		16	4	
N Stage				0.033*			0.299
N0	96	54	42		79	17	
N1	45	33	15		39	9	
N2	53	31	22		34	15	
N3	88	38	50		71	17	
Lymphovascular invasion				0.059			<0.001*
Negative	207	120	87		57	43	
Positive	65	29	36		166	16	

P values are based on a X2 test, *Significant difference

We found that the increased expression of flotillin2 and erbB2 was both significantly associated with histological type (p<0.001, respectively), Lauren grade (p<0.001, respectively) and Lymphovascular invasion (p = 0.059 and p<0.001, Table 2). In addition, flotillin2 was significantly correlated with tumor size (p = 0.006), T stage (p = 0.001) and lymph node metastasis (p = 0.033, Table 2). However, no significant correlation was observed between flotillin2 expression and other parameters including age, gender, location, macroscopic type and hepatic metastasis.

### Expression of flotillin2 in relation to prognosis

**Figure 3:**
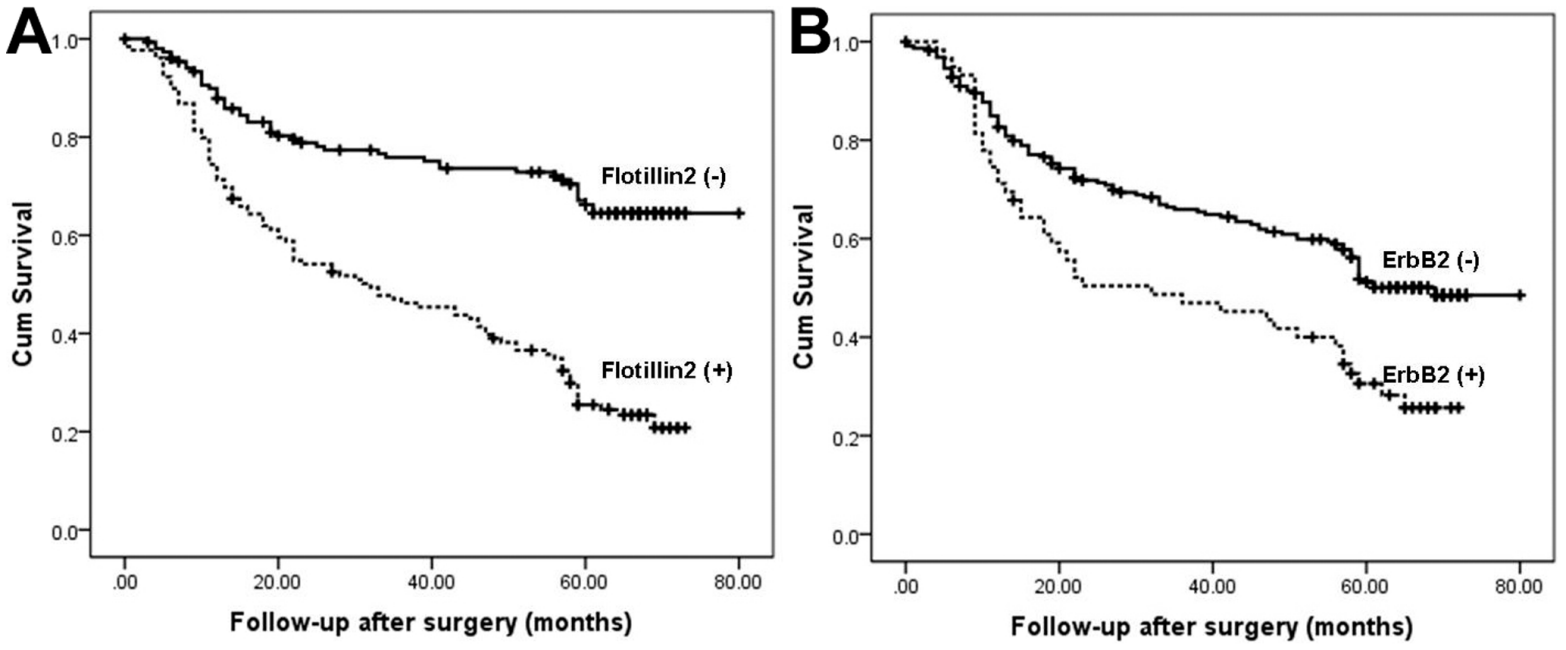
Kaplan-Meier curves for patient survival. (A) Patients with an erbB2-positive tumor showed a significantly shorter survival period after surgery than those with an erbB2-negative tumor (p  = 0.014). (B) Patients with a flotillin2-positive tumor showed the worst outcome. High flotillin-2 and erbB2 expression are significantly correlated with poor patient outcome (p<0.001).

Using Cox's proportional hazards regression model, the univariate relationships between tumor characteristics and patients' outcome were obtained (Table 3). Of the 282 patients analyzed, statistically significant differences in OS were seen, with a poor outcome for patients with higher staining of flotillin2 and erbB2. Other predictive factors that were found to be correlated with OS were age (P = 0.042), size (P = 0.001), macroscopic type (P = 0.001), pT (P<0.001) and pN stage (P<0.001) (Table 3). Using log-rank test, there were significant differences in OS between positive and negative patients of flotillin2 and erbB2 (P<0.001, respectively) in all patients were shown in Figure 3.

**Table 3:** Univariate analysis of prognostic variables in overall survival (OS) for patients.

Variables	Cases	5-year OS(%)	P value
Gender			0.351
Female	205	42.9	
male	77	50.2	
Age(years)			0.04*
≤65	182	52.7	
>65	100	40	
Size			0.001*
≤4 cm	135	60.7	
>4 cm	147	36.7	
Location			0.951
Lower	217	50.7	
Middle	21	42.9	
Upper	39	43.6	
Entire	5	36.5	
Macroscopic Type			0.001*
Early stage	31	87.5	
Borrmann I	4	50	
Borrmann II	20	80	
Borrmann III	199	44.7	
Borrmann IV	28	10.7	
Histologic type			0.134
Differentiated	124	53.2	
Undifferentiated	158	44.3	
Lauren grade			0.117
Intestinal	137	55	
Diffuse	145	43.1	
T Stage			<0.001*
T1	31	78.6	
T2	50	65.7	
T3	181	40.2	
T4	20	15	
N Stage			<0.001*
N0	96	72.9	
N1	45	54.2	
N2	53	46.9	
N3	88	19.3	
Lymphovascular invasion			0.001*
Negative	207	53.1	
Positive	65	32.3	
erbB2 Expression			<0.001*
Negative	223	53.4	
Positive	59	28.8	
Flotillin2 Expression			<0.001*
Absent	153	68	
Present	129	24.8	

* Significant difference.

Cox multivariate proportional-hazard regression model was then used to determine which factors were jointly predicative of OS. Variables which were thought to be significant in univariate analysis were included in the analysis. The significance, adjusted for other co-variates, was given in Table 4. Multivariate analysis of the joint effect of flotillin2 and erbB2 with other prognostic factors showed that pT (P<0.001) and pN stage (P<0.001) flotillin2 (HR  = 1.485, 95% CI: 1.278–1.725, P<0.001) was a better independently prognostic marker than erbB2 (HR = 1.375, 95%CI: 1.133–1.947 P = 0.004) for OS (Table 4).

**Table 4:** Multivariate analysis of overall survival after surgery.

Variables	5-year OS(%)	
	HR(95%CI)	P value
Age	1.334 (0.940–1.895)	0.107
Size	1.078 (0.747–1.556)	0.687
Macroscopic Type	1.434 (1.033–1.990)	0.031
T Stage	1.933 (1.344–2.781)	<0.001*
N Stage	1.397 (1.188–1.642)	<0.001*
Lymphovascular invasion	1.055 (0.719–1.548)	0.783
erbB2	1.375 (1.133–1.947)	0.004*
Flotillin2	1.485 (1.278–1.725)	<0.001*

HR hazard radio, CI confidence interval, *Significant difference.

## Discussion

The assessment of biological prognostic factors is of clinical importance, especially for a disease with poor outcome such as gastric cancer. The present study confirmed the constitutive expression of flotillin2 in gastric cancer tissue specimens and cell lines, we also found that flotillin2 was an independent poor prognostic factor of overall survival of gastric cancer patients. In addition, the present study showed, for the first time, a positive relationship between the expressions of flotillin2 and HER2 in gastric cancer. In the previous study, Pust et al. (2012) found that breast cancer cell lines derived from distant metastasis showed a higher level of flotillin mRNA and protein expressions than those derived from primary gastric cancer. Thus, they explained the role of flotillin2 as a tissue- and stage-specific tumor modulator, where it acts as an inhibitor or promoter of tumor formation and progression depending on its protein interaction partners such as growth factor receptors or cell adhesion molecules [11]. Our results agree with this description because flotillin2 expression in the present study was positively correlated with HER2 expression, which was positively correlated with poor prognosis in gastric cancer.

Data from two recent publications indicate that flotillin has a functional role in receptor tyrosine kinase signaling, especially in the activation of MAP kinase signaling and the regulation of FGF signaling in HeLa cells [16], [17]. In the SKBR3 breast cancer cells, the result that Akt phosphorylation is reduced upon depletion of flotillin-2, but not after flotillin-1 knockdown, agreed with our data. Thus, receptor tyrosine kinases or receptor tyrosine kinase-mediated signaling pathways might be regulated by flotillins in different ways and/or in a cell type-specific manner, it is possible that the different signaling pathways triggered by erbB2 are regulated by either flotillin2. Nevertheless, the reduced erbB2 levels triggered by flotillin2 knockdown support our hypothesis of a flotillin2-mediated stabilization of erbB2.

ErbB2 is known to be associated with poor prognosis in gastric cancer, flotillins have been described to be associated with tumor genesis. We demonstrated that flotillin2 co-localizes with erbB2 at the plasma membrane and plays a functional role on the regulation of erbB2 in gastric cancer. High expression of flotillin2 is associated with poor prognosis and reduced survival time, and it emerges as a potential predictor of relapse in gastric cancer. It has been reported that oestrogen receptors were involved in gastric carcinogenesis and flotillin2 interacts with the receptors [18], [19], indicating a significant impact of flotillin2 function on gastric cancer development. On the other hand, based on our tissue microarray studies, flotillin2 overexpression was highly associated with increased cell migration and malignant transformation leading to lymph node involvement and increased invasiveness. In our study, elevated flotillin2 and HER2 both showed a significant correlation with histologic type (P<0.001 both), Lauren grade (P<0.001 both), whereas flotillin2 also get a high relevance with T stage (P<0.001) and lymph node metastasis (P = 0.033). This strong correlation suggests that flotillin2 can be used as a biomarker to identify subsets of patients with gastric cancer with a more aggressive phenotype. The multivariate analysis demonstrated flotillin2 serves as a better prognostic marker to predict the risk of metastasis and recurrence (HR = 1.485, 95%CI: 1.278–1.725) than Her2 (HR = 1.375, 95%CI: 1.133–1.947) for OS. We propose that flotillin2 over-expression may contribute to increased proliferation and the development of gastric cancers.

In conclusion, this is the first demonstration of a positive correlation between flotillin2 and erbB2 levels in cultured gastric cancer cells as well as in tissues. Moreover, flotillin2 expression level correlates with poor prognosis in gastric cancer, indicating that flotillin2 might be a prognostic biomarker for gastric cancer. It has been found that erbB2-overexpressing tumors are more likely to be resistant to treatment with an antibody-based (trastuzumab/Herceptin) therapy, which has become the clinical first-line treatment in patients with erbB2-overexpressing metastatic gastric cancer [20], [21]. However, even in combination with other chemotherapeutical medications, more than 20% of patients with erbB2-overexpression show no response to the treatment. Thus, by targeting flotillin2 expression, new strategies in cancer treatment can be developed.
